# Micromycete diversity and pathogenic potential in soils of long-term cultivated apple orchards

**DOI:** 10.3389/fmicb.2025.1673468

**Published:** 2025-12-18

**Authors:** O. Demyanyuk, V. Oliferchuk, R. Yakovenko, D. Synenko, M. N. Coelho Pinheiro, L. Symochko

**Affiliations:** 1 Institute of Agroecology and Environmental Management NAAS, Kyiv, Ukraine; 2 National Forestry University of Ukraine, Lviv, Ukraine; 3 Uman National University of Horticulture, Uman, Ukraine; 4 Polytechnic University of Coimbra, Coimbra Institute of Engineering, Coimbra, Portugal; 5 CERNAS, Research Centre for Natural Resources, Environment and Society, Coimbra Agriculture School, Coimbra, Portugal; 6 CEFT, Transport Phenomena Research Center, Faculty of Engineering, University of Porto, Porto, Portugal; 7 Centre for Functional Ecology, University of Coimbra, Coimbra, Portugal; 8 Uzhhorod National University, Uzhhorod, Ukraine

**Keywords:** micromycetes, species diversity, mycobiome structure, *Malus domestica*, perennial fruit orchards, soil

## Abstract

**Introduction:**

Long-term agricultural management can substantially alter soil microbial communities. The vertical distribution and ecological roles of micromycetes in deep soil profiles of perennial orchard systems remain poorly understood. This study examines the abundance, taxonomic composition, and stratification of micromycetes in dark gray soils of apple orchards cultivated continuously for over 90 years, with the aim of identifying microbiological hotspots and assessing their potential ecological functions.

**Methods:**

Soil samples were collected from 0-100 cm depths and analyzed using standard microbiological methods. Quantitative assessments of fungal abundance were based on colony-forming unit (CFU) counts, while qualitative analysis included isolation and identification of micromycetes to the species level. Structural indices were calculated to characterize species diversity, community stability, and vertical differentiation across soil layers.

**Results:**

Micromycete abundance remained consistently high throughout the soil profile, ranging from 113 to 138 × 10^3^ CFU g^−1^, indicating persistent fungal activity across depths. A total of 68 species belonging to 22 genera and three phyla (*Mucoromycota*, *Mortierellomycota*, and *Ascomycota*) were identified. *Ascomycota* dominated the mycobiome, accounting for 85% of species diversity. *Aspergillus* (14 species) and *Penicillium* (13 species) were present at all depths, suggesting their central role in shaping microbial hotspots. Rare taxa, such as *Mucor hiemalis*, *Cladosporium cladosporioides*, and *Humicola* spp., occurred at low frequencies (0.3–3.4%), contributing to community heterogeneity. Importantly, *Fusarium culmorum*, typically associated with chernozem soils, was detected for the first time in dark gray soils at 20–60 cm depths. Structural indices revealed clear stratification between surface and subsurface horizons, with greater species richness and community stability observed in the 0-60 cm layers.

**Discussion:**

These findings demonstrate that long-term orchard cultivation supports vertically structured micromycete communities, with specific soil layers acting as microbiological hotspots essential for maintaining soil ecosystem functions. The substantial proportion of phytopathogenic taxa (28%) underscores potential risks to orchard health, while the novel detection of F. culmorum suggests shifts in fungal distribution driven by prolonged land use.

## Graphical abstract

**Figure:**
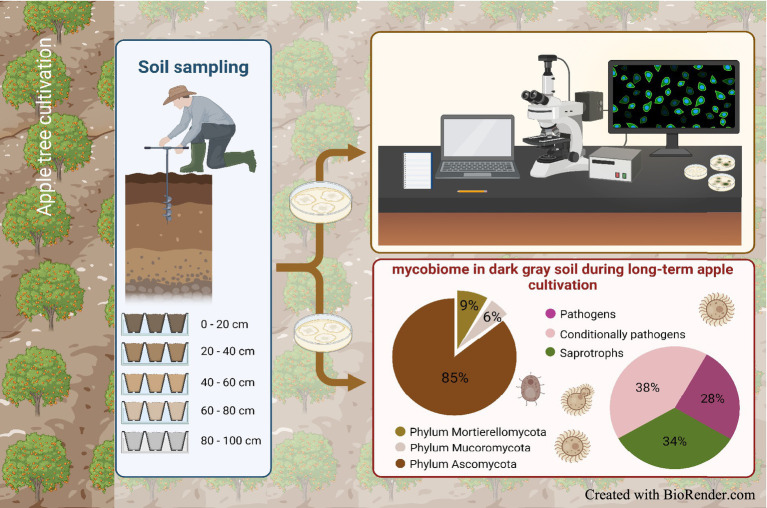

## 1 Introduction

Soils and the biodiversity associated with them are integral and essential components of terrestrial ecosystems, playing a key role in delivering a wide range of ecosystem services and maintaining soil health (Bardgett and Van Der Putten, 2014; Laban et al., 2018; Symochko et al., 2018; Brondizio et al., 2019; Wagg et al., 2019). Among soil biodiversity, bacteria and fungi play direct and indirect roles in many fundamental ecological functions, including nutrient cycling, synthesis and decomposition of organic matter, and carbon sequestration, all of which ensure soil quality and health (Paul, 2016; Laban et al., 2018; Demyanyuk et al., 2019; Demyanyuk et al., 2020a). Additionally, the soil microbiome directly influences plant health through symbiotic and pathogenic interactions (Bardgett and Van Der Putten, 2014; De Coninck et al., 2015; Symochko et al., 2021; Zandt et al., 2023). It is well known that soil is the foundation for agricultural production. The effectiveness of various agricultural practices largely depends on the physical, chemical, and biological properties of the soil, which ultimately determine the productivity of crops, the profitability of farming, and food security. However, the ecological state of the soil, determined by biodiversity, biological activity indicators, the direction of microbiological processes, the structure of the microbiome, dominant microorganism species, and the abundance and species composition of pathogenic microbiota, is often overlooked. In-depth studies of not only the physical–chemical and agrochemical properties of soil in agroecosystems but also microbial communities can serve as the basis for developing and implementing the most effective and environmentally sustainable soil management methods, helping to create sustainable agroecosystems that meet the food demands of a growing global population. This issue is especially relevant for perennial fruit orchards, which are monoculture agrobiocenoses, where the phenomena of soil fatigue and specific replant disease (SRD) are likely to occur (Manici et al., 2013; Winkelmann et al., 2019; Yakovenko et al., 2023). The soil environment is the most complex and diverse reservoir of biological species in the biosphere, including bacteria and fungi (Mendes et al., 2013; Laban et al., 2018; Brondizio et al., 2019; Tibbett et al., 2020; Symochko et al., 2023). Advances in modern molecular-genetic methods continue to expand our knowledge of soil microbiota, their properties, and significance (Anderson and Cairney, 2004; Peay et al., 2016). The significant species diversity, abundance, and physiological-biochemical properties of soil microorganisms make them valuable bioindicators of various ecological and anthropogenic factors, as well as tools for assessing soil health (Demyanyuk et al., 2018; Fierer et al., 2021). An important component of soil microbial communities are micromycetes, whose primary contribution to ecosystem functioning is related to soil stabilization, fertility formation, participation in the natural cycles of carbon, nitrogen, phosphorus, and other elements, decomposition of organic matter, humus synthesis, pollutant breakdown, and more. These processes directly and indirectly affect the quality and health of both soil and plants (Christensen, 1989; Schloter et al., 2003; Wagg et al., 2019; Demyanyuk et al., 2020b). Additionally, some micromycete species, due to their specific properties, can act as biocontrol agents, stimulate plant growth and development, and counteract phytopathogens. On the other hand, some micromycetes can be harmful to plants, inhibiting their growth and development (van der Heijden and Hartmann, 2016; Aslam et al., 2017; Kopylov and Nadkernichna, 2017; Boldt-Burisch et al., 2023; Nikitin et al., 2023). It is known that approximately 70% of plant infectious diseases are caused by fungi (Li et al., 2022). Research has shown that agricultural practices (such as soil tillage, fertilization, and the use of pesticides), along with other factors, significantly influence the taxonomic composition, abundance, and metabolic activity of microbial communities, including the mycobiome (Orgiazzi et al., 2016; Yang et al., 2021). There is growing evidence that agricultural practices and climate change are leading to a loss of soil biodiversity in agroecosystems, simplifying the structure of the microbiome, including the bacteria-to-fungi ratio, making it less complex and resilient than the microbiomes of natural ecosystems (Creamer et al., 2016; Santos et al., 2020; Wagg et al., 2020; Lammel et al., 2021). For instance, the intensification of agriculture reduces the complexity of root fungal networks and the number of key taxa (Banerjee et al., 2019). In agroecosystems, understanding the composition and structure of the soil mycobiome is crucial for better comprehending the functions of microbial communities, the ecological state of soils, and managing agroecosystems more effectively (Landinez-Torres et al., 2019). This knowledge is especially important for soil conservation, fertility restoration, carbon sequestration, reducing soil phytotoxicity, and improving the productivity of field and perennial fruit crops. Studying the soil mycobiome is also highly relevant in cases of continuous monoculture farming, including fruit (orchard) agrobiocenoses. Such research is ongoing in various agrobiocenoses to deepen our understanding of soil biodiversity. For instance, studies in pear orchards have identified 35 fungal species, including several species of Mortierella, Humicola, Solicoccozyma, and Exophiala, with 79% of the identified fungal species being recorded for the first time (Nicola et al., 2021). It has been found that peach and apple orchards contain relatively small groups of fungal species belonging to three main phyla: Ascomycota, Basidiomycota, and Zygomycota, with the genus Mortierella representing the largest number of species (Landinez-Torres et al., 2019). In the overall soil microbiome of the apple orchard, the most prevalent fungi were from the phyla Ascomycota, Basidiomycota, Mortierellomycota, and Glomeromycota, accounting for 80, 13, 5, and 1%, respectively. In contrast, within the soil mycobiome of the apple orchard, representatives of Ascomycota (67%), Basidiomycota (22%), and Mortierellomycota (10%) were most common, including classes such as Sordariomycetes, Leotiomycetes, and Dothideomycetes from Ascomycota, and Tremellomycetes from Basidiomycota, which were represented by 29, 16, 14, and 20%, respectively, (Ajeethan et al., 2023). It has been shown that perennial woody crops, such as apple trees, develop more stable interactions with microbial communities in the rhizosphere due to the relatively long lifespan of perennial plants and the absence of soil disturbances like annual crop rotation (Mercado-Blanco et al., 2018). Studies of the relationships between bacterial, archaeal, nematode, and fungal communities in the root zone of 10-year-old apple orchards revealed complex associations within the microbiome. These studies demonstrated diversity and structure typical of perennial crops, with high diversity, high evenness, and many rare species (Bintarti et al., 2020). It is well known that monoculture disrupts the biological balance between saprophytic and pathogenic microbiota, favoring the accumulation of the latter (Sobiczewski et al., 2018; Liu et al., 2021; Zhang et al., 2023). The main reason for changes in microbial communities in monocultures is the prolonged accumulation of homogeneous plant residues and root exudates, including phenolic acids or phytotoxins (Narwal et al., 2005; Bai et al., 2009; Weib et al., 2017; Manici et al., 2018), which can inhibit plant growth and promote the increase of phytopathogenic species responsible for root diseases and producers of phytotoxic substances. For example, the most pathogenic fungi from the genera *Cylindrocarpon* and *Rhizoctonia* are frequently found in the soil of perennial apple orchards, along with oomyces *Phytophthora* and *Pythium*, which are recognized as key agents in the development (etiology) of apple replant disease (ARD) (Manici et al., 2013; Manici et al., 2018; Ajeethan et al., 2023). This is a global problem that occurs in various apple-growing regions and across different soil types, yet its causes remain unclear (Winkelmann et al., 2019). In the soil of apple orchards, the pathogenic microbial complex often includes micromycetes such as *Fusarium* species (*F. oxysporum, F. solani, F. equiseti, F. proliferatum*), *Verticillium*, as well as *Rhizoctonia solani* (teleomorph: *Thanatephorus cucumeris*), *Cylindrocarpon*, and others, which infect tree roots and cause wilting, leading to significant economic losses (Jiang et al., 2017; Sharma and Marques, 2018; Ajeethan et al., 2023; Sharma et al., 2023). In particular, the negative impact of phytopathogenic strains of *Fusarium* is associated with global economic losses in agriculture worldwide, amounting to billions of U. S. dollars annually (Johns et al., 2022). Two species, *F. graminearum* and *F. oxysporum*, are among the top five major fungal plant pathogens (Ploetz, 2006; Sharma and Marques, 2018; Bakker et al., 2018; Timmusk et al., 2020; Karlsson et al., 2021). Data on the distribution of various *Fusarium* species in apple orchard soils worldwide and their role in the development of ARD (Apple Replant Disease) is well documented (Duan et al., 2022; Jiang et al., 2022). The increase in the proportion of *Verticillium* species in the microbiome under long-term continuous apple cultivation suggests their involvement, among other bioagents, in the development of ARD (Jiang et al., 2017). At the same time, researchers note that ARD is not solely related to the increased number of pathogens but likely to changes in the composition of the soil microbiota (Radl et al., 2019; Xu et al., 2023). This underscores the need to study soil microbiomes and their changes based on soil-climatic conditions and agricultural practices. Microfungi in the soils of perennial fruit plantations are also studied to improve soil health by selecting beneficial species for cultivation in laboratory conditions and their subsequent introduction into the soil or compost (Chaturvedi et al., 2013). This bioremediation and enrichment of the soil mycobiome with native strains is crucial as it aims to maintain the balance of soil microfungal biodiversity within a particular agrobiocenosis (Da Silva et al., 2015; Sethuraman et al., 2020). Thus, considering the above, studying the mycobiome of soils in perennial fruit plantations, including species diversity, structure, and phytopathogenic complexes, is relevant not only for achieving high yields but also for ensuring the biological safety of agrobiocenoses. The objective of the research is to study the species structure and abundance of microfungi in the soil under long-term (over 90 years) apple cultivation.

## 2 Materials and methods

**Figure 1:**
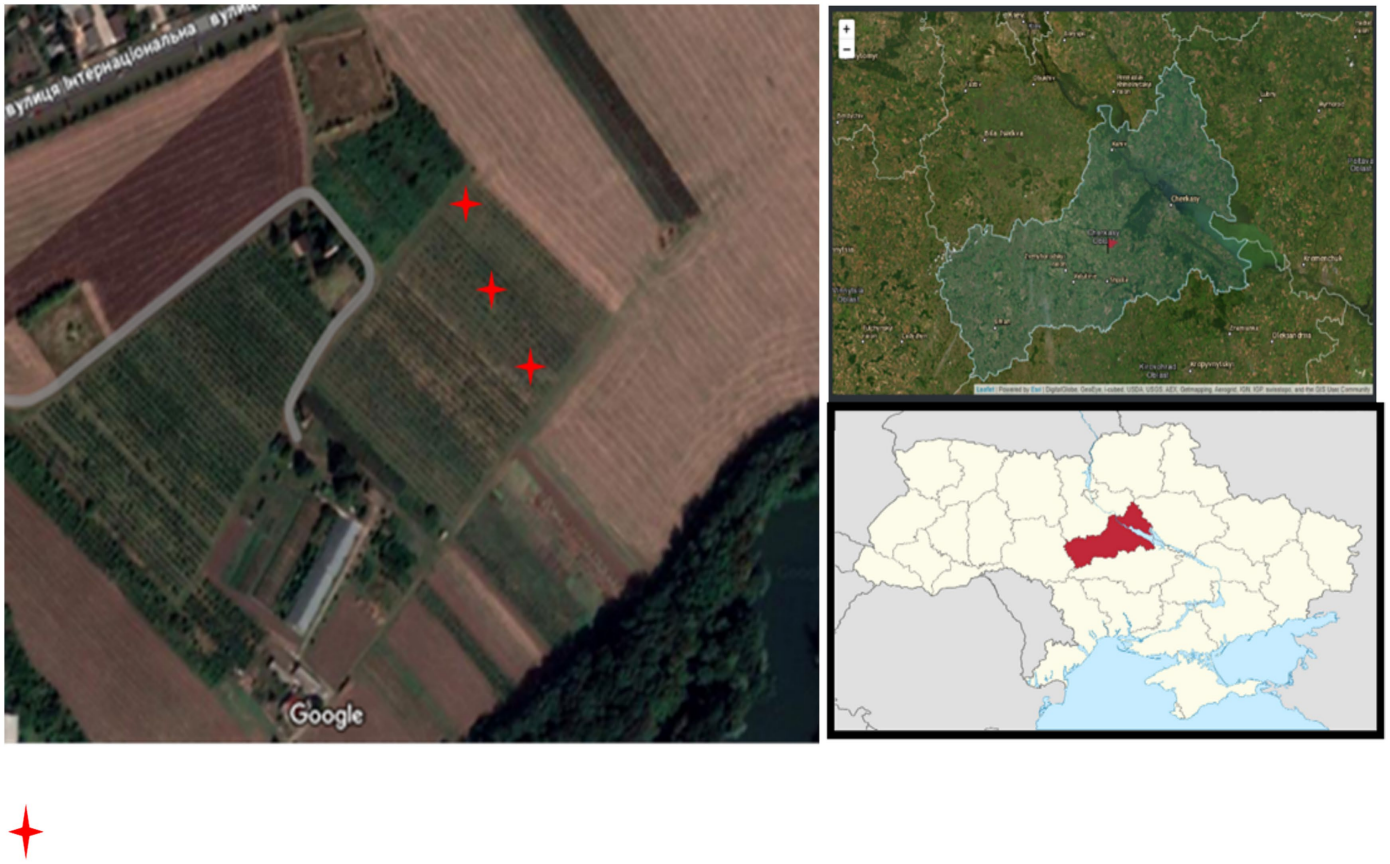
The map of the Cherkasy region, Ukraine, shows the study area and a satellite image from Google Earth with the study sites superimposed (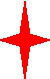 —soil sampling plots).

The study of the species structure and abundance of soil microfungi was conducted in long-standing apple orchards at the Uman National University of Horticulture, located in the Cherkasy region (geographical coordinates 48.761119 N, 30.251359E) (Figure 1). These orchards were chosen primarily because they represent an area considered to be a prime location for orchards in Ukraine, where apples have been grown for many decades on family farms, farming enterprises, and large horticultural companies. Similar old apple orchards (over 30–40 years old) can be found in the Right-Bank Forest-Steppe region of Ukraine, particularly in the Cherkasy and Vinnytsa regions. These orchards are in urgent need of reconstruction, and new intensive orchards will be established in these areas in the coming years.

The orchard was planted in 1931 with Calville Snow apple trees variety on seedling rootstock, planted at 10 × 10 meters and maintained for 50 years. In 1982, the orchard underwent a reconstruction, during which the old trees were uprooted. In 1984, new trees were planted, including Calville Snow on seedling rootstocks and Idared on both seedling and vegetative M4 rootstocks, with a planting distance of 7 × 5 meters, while preserving the original research plot layout. A second reconstruction took place in 2017, when the trees were uprooted again, and new trees were planted in 2018. This time, Golden Delicious and Gala varieties were introduced in MM.106 rootstock, with a planting distance of 5 × 2 meters, maintaining the same research plot layout. Throughout the orchard, the inter-row spaces are managed with black fallow, and herbicide fallow (Roundup Max, active ingredient: glyphosate potassium salt, 551 g l^−1^) is used to maintain the tree rows. The experimental orchard is not irrigated. Pest and disease management in the orchards follows a chemical protection system, using products at the levels recommended by the manufacturer: Іnsecticides (Aktara 25 WG – active substance Thiamethoxam, 250 g·kg^−1^; Konfidor Maksi, WG—active substance Imidacloprid, 700 g·l^−1^; Mospilan – active substance Acetamiprid, 200 g·kg^−1^; Match 050 EC – active substance Lufenuron, 50 g·l^−1^; Liufoks 105 ЕС – active substance Fenoxycarb, 75 g·l^−1^ + Lufenuron, 30 g·l^−1^). Fungicides (Kosaid 2000 wg – active substance copper hydroxide, 538 g·kg^−1^; Skor 250 EC—active substance Difenoconazole, 250 g·l^−1^; Topsin М, sp.—active substance Thiophanate-methyl, 700 g·kg^−1^; Akira, SС—active substance Captan, 370 g·l^−1^; Manzat, WG—active substance Mancozeb, 750 g·kg^−1^; Delan, WG—active substance Dithianon, 700 g·kg^−1^). Herbicides (Raundap Maks, SL—active substance Glyphosate Potassium Salt, 551 g·l^−1^).

The characteristics of the dark gray podzolic soil are detailed in Table 1.

**Table 1:** Physico-chemical characteristics of the greyzemic phaeozems at the sampling sites.

Parameter	Depth (cm)	
	0–20	20–40
pH ± 0.10	5.2	5.3
Organic matter (%)	2.41	2.23
N_tot_ (g·kg^−1^ SS)	13.4	12.9
C_org_ (% SS)	56	53
C/N	4.2	4.1
P (mg·kg^−1^ SS)	184	146
Ca (meq/100 g)	18.4	20.1
Mg (meq/100 g)	4.32	3.95
K (meq/100 g)	28.9	27.4
Sand (%)	18.0	17.4
Silt (%)	30.6	29.6
Clay (%)	51.4	53.0

Soil samples for mycological studies were collected from layers of 0–20 cm, 20–40 cm, 40–60 cm, 60–80 cm, and 80–100 cm layers following international standards (ISO, 2018). Soil samples were collected from three plots, each of which had three sampling squares (10 m × 10 m). Each square contained five sampling points: one at each corner and one in the center. A minimum distance of 3 m from the tree was maintained. Soil samples were taken from five layers at 20 cm depth intervals (0–100 cm). Samples from the same soil layer were then combined to form a single composite sample and labeled according to soil depth for each square. All samples were stored in a sampling container with ice packs.

The quantitative and qualitative composition of micromycetes was determined using a standard microbiological method. One gram of the examined soil was diluted in 10 mL of sterile water, and 10^−3^ and 10^−4^ dilutions were used for plating on wort-agar. One milliliter of the suspension was spread over the surface of the wort-agar using a sterile spatula and incubated at 28 °C for 4 to 14 days. All single-factor experiments were performed in triplicate.

The morphology of the fungi was examined using a MICROmed XS-3330 LED light microscope at magnifications of ×200, ×320, and ×400, after calculating the value of the ocular-micrometer scale for each magnification. Micromycete identification was carried out based on an integrated approach that combined macro- and micromorphological analysis, cultural characteristics, and comparison with authoritative taxonomic keys. Cultural characteristics - including colony color, texture, edge morphology, degree of sporulation, and pigment production were recorded after standard incubation periods. Microscopic examination included assessment of hyphae structure, conidiophores, conidia size and shape, septation, ornamentation, and arrangement of reproductive structures. Measurements of conidia and other diagnostic structures were taken using an ocular micrometer to ensure accurate species-level differentiation. The identification of each isolate was finalized through comparison with classical and modern mycological reference guides, widely recognized for soil micromycete taxonomy (Barnett and Hunter, 1998; Domsch et al., 2007; Watanabe, 2010). Genus and species nomenclature were updated to align with modern taxonomic systems and databases such as MycoBanck.^1^ The species’ percentage occurrence was determined based on sampling depth. Biodiversity across the soil layers was assessed using *α*-diversity indices calculated with Mothur software (version v.1.30.2).^2^ Species diversity within micromycete communities was characterized using diversity and evenness indices commonly applied in general ecology, which quantify the relationship between species richness and abundance. To assess micromycete diversity, frequency and abundance were measured, community similarity was calculated using Sorensen’s coefficient, and species diversity was assessed using Shannon’s index. Additionally, Simpson’s index and Pielou’s evenness index were applied to evaluate species dominance within the communities. A Venn diagram was created to visualize the overall soil mycobiome, highlighting species unique to the sampling sites and those shared among them (Heberle et al., 2015).^3^ Species from the phyla *Mucoromycota, Mortierellomycota* and *Ascomycota* with an abundance below 0.5% were excluded.

The experimental results were statistically analyzed using the Statistica 10 software. The tests were performed in 3–5 repetitions. Mean values (x) and their standard deviations (SD) were determined. The level of significance selected for the study was *p* < 0.05. Dispersion analysis and the Tukey test were used to compare the averages of the independent samples. Logistic transformation was applied to the data and expressed as a percentage.

## 3 Results

After more than 90 years of apple cultivation, the total number of micromycetes in the soil ranged from 113 to 138 × 10^3^ CFU/g of soil (Table 2). The high abundance and species diversity of micromycetes in the upper soil layers can be attributed to the high organic matter content and the concentration of tree roots in these layers.

**Table 2:** Abundance of micromycetes and α-diversity indices in different soil layers during long-term apple cultivation.

Indicator	Soil layer (cm)				
	0–20	20–40	40–60	60–80	80–100
Number of propagules (×10^3^ CFU/g of soil)	121 ± 8	138 ± 13	113 ± 5	121 ± 8	125 ± 9
Number of isolated species	51	54	35	20	14
*α*-diversity indices					
Shannon–Weaver	2.32	2.53	1.37	1.16	1.02
Pielou	0.68	0.71	0.48	0.34	0.21
Simpson	0.056	0.061	0.031	0.012	0.009
Sørensen–Czekanowski	9.24	9.32	8.11	4.56	3.64
Berger–Parker	5.7	6.2	4.9	3.9	2.7

Structural indicators of micromycete communities at different depths show significant differences between the surface and deeper soil layers. Species diversity indices are higher in the surface layers (0–20 cm, 20–40 cm, and 40–60 cm), indicating greater stability of micromycete community structures at these depths. Similarly, the Shannon, Simpson, and Pielou indices are also higher in these surface layers. The highest species diversity was observed at a depth of 20–40 cm, suggesting the formation of stable micromycete complexes in this layer.

In deeper soil layers, the species composition of the micromycetes shows an improvement, as indicated by a 2.3-fold decrease in the Berger-Parker index. The species diversity of micromycetes increases from the plant cover to the litter and the upper mineral horizon. These patterns are consistent with the functional traits of micromycetes, such as the production of a wide range of hydrolytic and other enzymes that allow them to utilize different compounds, including those that are difficult for other microorganisms to access, filamentous growth that allows colonization of various substrates, xerophytic capacity, and thermotolerance.

The surface soil layers exhibit higher values for species richness, diversity, and evenness with minimum values recorded at depths of 60–80 cm and 80–100 cm. Detailed analysis of the ecotopes showed that the Simpson index decreased with depth and remained higher in the surface layers, suggesting a more balanced dominance structure. The high index values in the surface layers are associated with a greater fungal diversity. The Pielou index, which measures species evenness, indicates the most even distribution of species at a depth of 20–40 cm, with almost identical values observed for the 0–20 cm and 20–40 cm depths.

**Figure 2:**
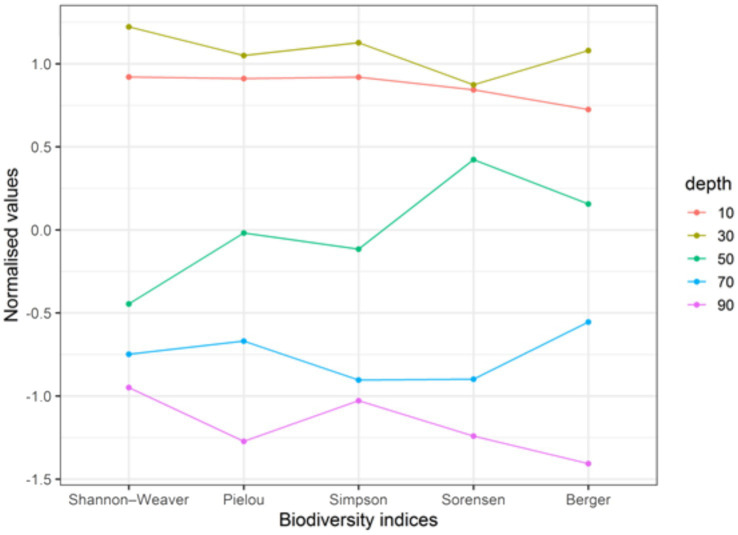
Distribution of normalised values of micromycete diversity indices in different soil layers (depth (cm) – median values of soil layers) (R Core Team, 2020).

To assess the similarity between ecotopes, the Sørensen-Czekanowski similarity index was applied, revealing differences in species diversity between the surface and deeper layers. Figure 2 shows the profiles of normalized micromycete diversity index values at different soil depths.

The diversity indices of the upper layers (0–20 cm and 20–40 cm) were significantly higher than those of the deeper layers. The 20–40 cm layer showed the highest micromycete species diversity, where the conditions for fungal growth and development are most favorable. All diversity indices exhibited a clear trend of decreasing mycobiota diversity with depth, as evidenced by the non-overlapping profiles. The deepest layer (80–100 cm) had the lowest diversity, with the smallest number, diversity, and biomass of species. Based on the indicators studied (diversity index and dominance index), two types of soil layers were identified:

1) Low diversity index values with a balanced dominance structure - observed in the 60–80 cm and 80–100 cm soil layers.
2) High diversity index values with a balanced dominance structure and more even species distribution—present in the 0–60 cm depths.

**Figure 3:**
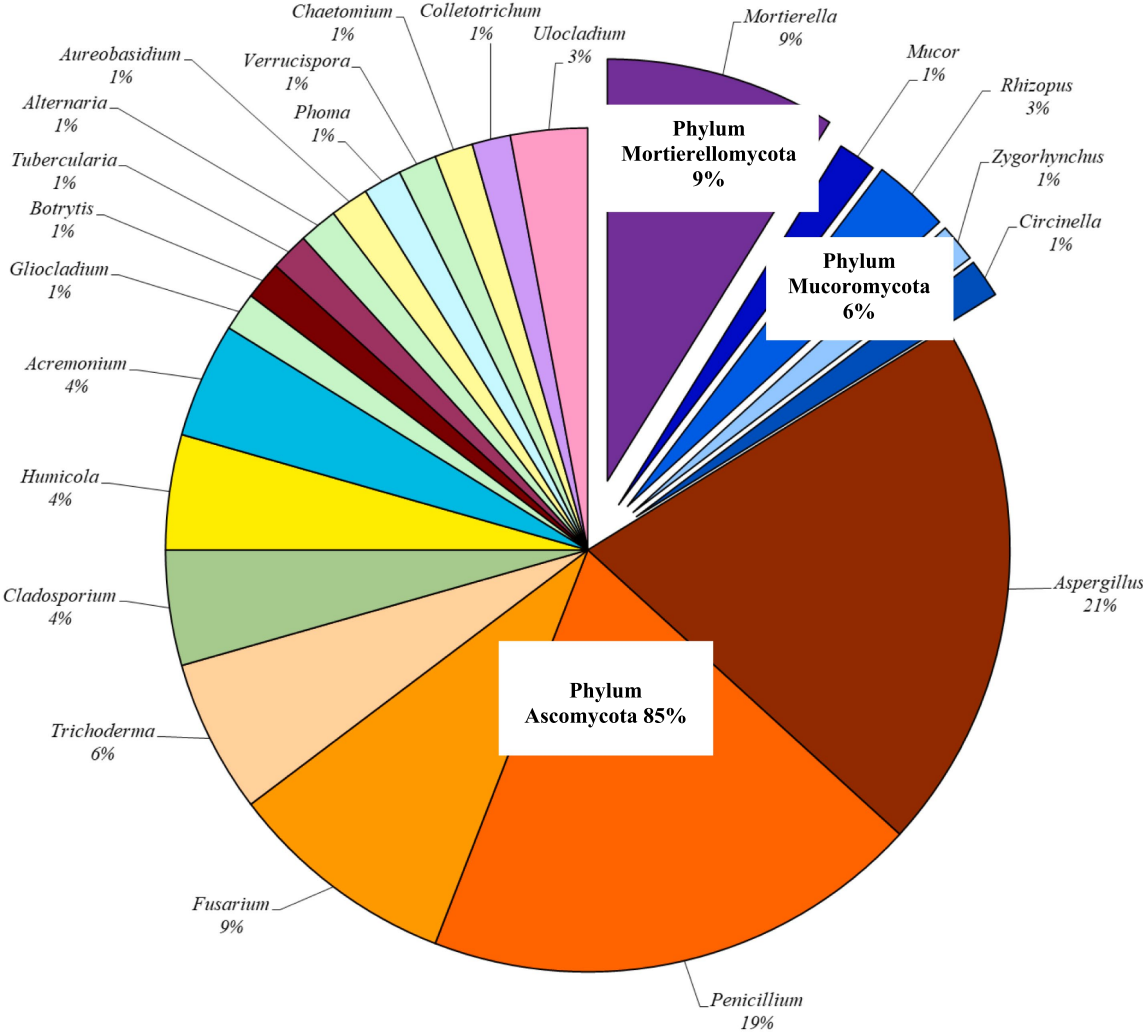
Overall structure of the mycobiome in dark gray soil during long-term apple cultivation, expressed as a percentage of the total number of isolated species (% of species relative to the total number of species isolated).

It was found that in the old orchard soil, the species diversity of micromycetes decreases with depth, although the number of fungal propagules remains relatively constant across layers. This stable and consistently high number of micromycetes throughout the soil profile suggests that stable complexes of soil micromycetes have formed during the long-term monoculture (over 90 years). A total of 68 species of micromycetes were isolated from the studied soil, belonging to 22 genera from three phyla: *Mucoromycota* (4 genera), *Mortierellomycota* (1 genus) and *Ascomycota* (17 genera) (Figure 3). Data on the genera and species of micromycetes isolated from the soil are presented in Table 3.

**Table 3:** Frequency of occurrence (%) of micromycete species at different soil sampling depths (cm).

№ п/п	Taxon name	Soil sampling depth (cm)				
		0–20	20–40	40–60	60–80	80–100
Subkingdom *Mucoromyceta*						
phylum *Mucoromycota*						
1	*Circinella umbellatа*	1.9	-	5.7	-	-
2	*Mucor hiemalis*	-	-	25.2	-	-
3	*Rhizopus oryzae* (syn. *Rhizopus arrhizus*)	-	1.7	-	-	-
4	*Rh. nigricans* (syn. *Rhizopus stolonifer* var. *stolonifera*)	-	1.2	-	-	-
5	*Zygorhynchus moelleri*	2.3	5.7	4.3	-	-
phylum *Mortierellomycota*						
6	*Mortierella alpinа*	1.2	5.3	0.4	-	-
7	*M. camargensis*	1.2	5.3	-	-	-
8	*M. elongata*	-	1.7	-	-	-
9	*M. exigua*	1.6	4.2	-	-	-
10	*M. hyalina*	-	1.5	1.3	-	-
11	*M. ramanniana* var. *ramanniana* (syn. *Umbelopsis ramanniana*)	0.7	2.6	-	-	-
Subkingdom *Dikarya*						
phylum *Ascomycota*						
12	*Acremonium kiliense*	8.5	-	-	0.5	-
13	*А. murorum* (syn. *Gliomastix murorum*)	4.7	-	-	-	-
14	*А. strictum* (syn. *Sarocladium strictum*)	5.4	1.2	-	-	-
15	*Alternaria alternatа*	2.5	12.5	0.5	-	-
16	*Aspergillus alliaceus*	5.0	1.2	-	-	-
17	*А. amstelodami*	7.0	1.3	-	-	-
18	*А. awamori*	3.2	5.7	3.1	-	-
19	*А. flavipes*	-	4.2	0.3	-	-
20	*A. flavus*	-	1.3	-	1.2	0.5
21	*A. fumigatus*	-	-	9.5	1.9	27.5
22	*A. niger*	-	39.0	13.7	18.0	56.0
23	*A. ochraceus*	1.0	4.0	0.5	-	-
24	*А. repens* (syn. *Aspergillus reptans*)	3.2	5.4	-	-	-
25	*А. ruber* (syn. *Eurotium rubrum*)	0.5	0.5	-	-	-
26	*А. sydowii*	1.8	1.3	-	-	-
27	*A. terreus*	1.8	-	-	11.0	23.0
28	*A. ustus*	1.4	-	-	13.0	-
29	*A. wentii*	7.0	-	-	7.2	-
30	*Aureobasidium pullulans*	10.7	7.6	-	-	-
31	*Botrytis cinerea*	-	5.2	1.7	-	-
32	*Chaetomium homopilatum* (syn. *Humicola homopilata*)	2.0	2.0	-	1.5	-
33	*Cladosporium cladosporioides*	-	3.4	3.2	11.0	-
34	*Сl. herbarum*	12.5	7.5	0.5	-	-
35	*Cl. sphaerospermum*	7.8	1.5	-	-	-
36	*Colletotrichum gloeosporioides*	-	1.5	-	-	2.4
37	*Fusarium lateritium*	2.3	-	-	-	-
38	*F. oxysporum*	2.3	2.3	1.0	0.5	0.5
39	*F. proliferatum*	-	-	0.7	-	-
40	*F. sambucinum*	1.7	0.5	-	-	-
41	*F. solani* (syn. *Neocosmospora solani*)	0.2	1.0	0.5	0.5	-
42	*Fusarium* spр.	1.7	0.5	0.5	0.5	0.5
43	*Gliocladium roseum* (syn. *Clonostachys rosea*)	7.5	1.7	-	-	-
44	*Humicola grizea* (syn. *Trichocladium griseum*)	3.4	0.3	0.3	-	-
45	*H. olivacea*	5.7	1.5	-	-	-
46	*Humicola* spр.	7.8	0.3	-	-	-
47	*Pеnicillium chrysogenum*	5.8	7.4	-	-	-
48	*P. citrinum*	2.7	4.3	0.5	-	-
49	*P. cyclopium*	11.0	-	1.4	15.0	-
50	*P. lanosum*	12.0	-	1.2	12.7	0.7
51	*P. luteum* (syn. *Talaromyces luteus*)	-	1.7	3.8	0.8	8.9
52	*P. multicolor*	-	4.0		0.5	-
53	*P. notatum* (syn. *Penicillium chrysogenum*)	-	6.8	-	-	12.7
54	*P. pallidum*	7.8	-	0.9	8.9	10.4
55	*P. purpurogenum* (syn. *Talaromyces purpureogenus*)	10.2	5.7	1.2	-	0.5
56	*P. spinulosum*	1.0	2.3	0.5	-	-
57	*P. tardum*	8.5	1.2	0.5	-	-
58	*P. variabile* (syn. *Talaromyces variabilis*)	2.3	1.0	-	1.0	-
59	*Pеnicillium* sрp.	4.5	1.0	1.7	0.5	0.5
60	*Phoma exigua* (syn. *Boeremia exigua*)	1.0	5.2	1.7	-	-
61	*Trichoderma atroviride*	11.6	7.5	4.5	-	0.5
62	*T. harzianum*	21.7	24.0	7.5	-	-
63	*T. koningii*	24.7	23.0	17.0	-	-
64	*T. viride*	32.0	23.0	20.0	-	-
65	*Tubercularia* spр.	0.1	7.1	-	-	-
66	*Ulocladium* spр.	2.7	7.3	0.3	-	-
67	*U. consortiale* (syn. *Alternaria consortialis*)	-	1.5	0.7	0.5	-
68	*Verrucispora proteacearum* (syn. *Zasmidium proteacearum*)	3.4	-	-	-	-

Table 4 shows the number of common micromycete species in different soil layers. The diagonal element is the maximum, as it represents the total number of species in that layer. The layers are marked with median depth values. The matrix is symmetrical, so only part of it is shown. The larger the value of the off-diagonal element, the greater the similarity in species composition between soil layers. The calculations were conducted based on the lists of micromycete species, without taking into account their frequency of occurrence.

**Table 4:** The number of common micromycete species at different depths of soil samples. (Diagonal elements are the total number of species in a soil sample of the corresponding depth).

Depth*	X10	X30	X50	X70	X90
X10	51				
X30	40	54			
X50	25	28	35		
X70	13	12	12	20	
X90	8	10	10	10	14

* Depth – median values of soil layers, in cm.

**Figure 4:**
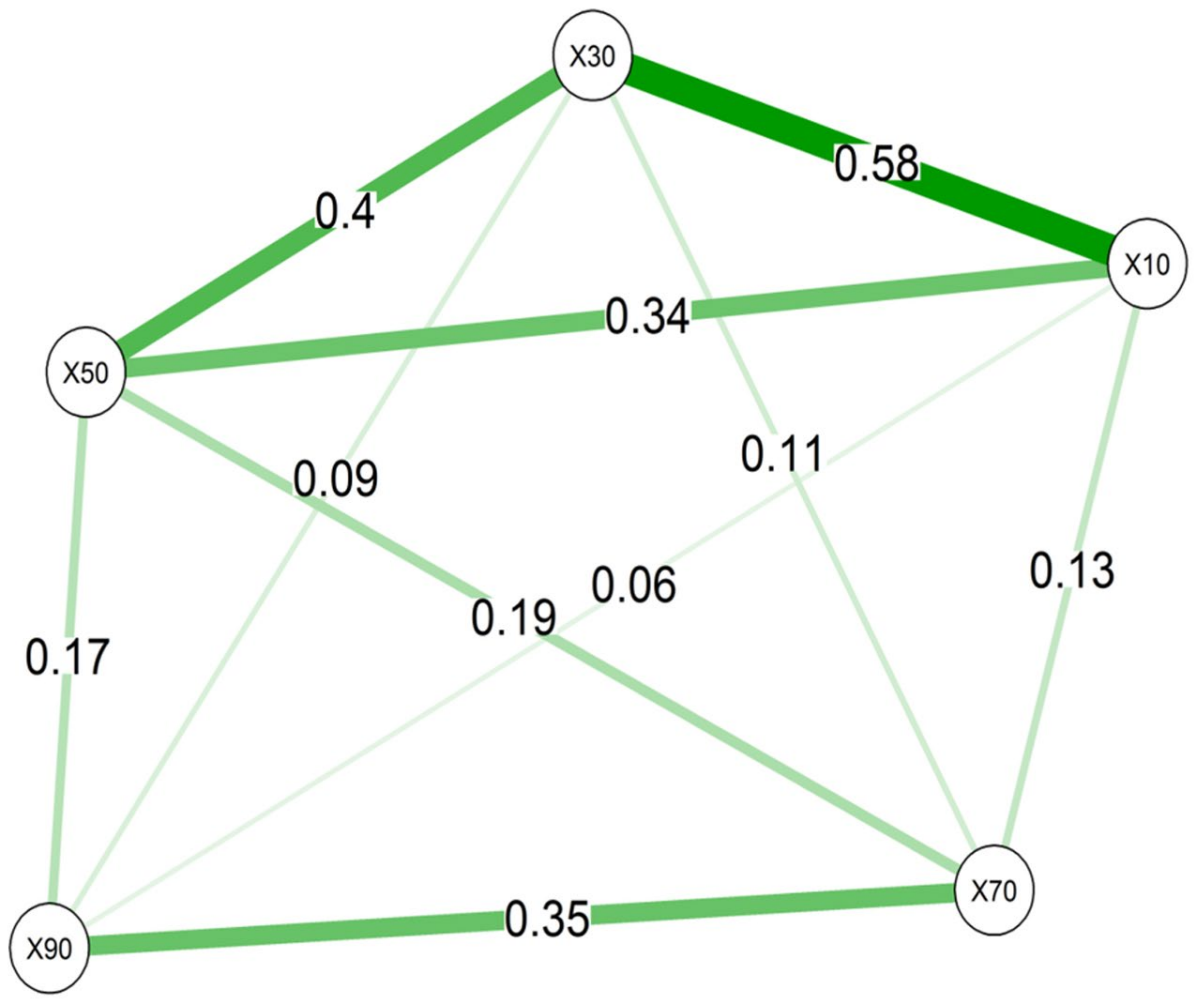
Graph of similarity in the species composition of micromycetes across different soil layers. The thickness of the edges represents the similarity values, with thicker edges indicating greater similarity. The nodes correspond to soil layers and their positions reflect the correlation structure of the data, with nodes closer together having more similar species compositions. The depth values in the nodes indicate the median depth of the soil layers (in cm) (R Core Team, 2020).

The greatest number of micromycete species was observed in the second layer (20–40 cm), with a similar number found in the first layer (0–20 cm). A clear trend of increasing differences in the species composition was observed with increasing depth and distance between layers, resulting in a lower number of common species with increasing depth. A significant similarity in species composition was observed between the first two layers. Figure 4 illustrates the similarity in species composition of micromycetes across different soil layers, with the greatest similarity between the 0–20 cm layer and the 20–40 cm layer. Beyond these layers, the similarity in species composition decreased markedly with increasing depth.

The study also showed that the species diversity of pathogens responsible for apple tree root diseases (rotting/decay) included seven species from six genera. The most common pathogens of apple root rot in the whole sample were species of the genus *Fusarium* spp. In addition, soil micromycetes varied between the different layers. In particular, species such as *Aspergillus niger*, *Colletotrichum gloeosporioides*, *Aspergillus fumigatus*, *Botrytis cinerea*, *Fusarium oxysporum*, *Alternaria alternata*, and *Cladosporium cladosporioides* were associated with root rot in apple seedlings and young trees. This is a potential risk when restoring old orchards and when planting new seedlings.

Similarity analysis of micromycete species composition across soil layers showed that the 0–20 cm and 20–40 cm layers had the highest degree of similarity, with a value of 0.58, indicating a substantial overlap in species composition between these upper layers. In contrast, the deeper layers, particularly the 60–80 cm and 40–60 cm layers, showed significantly lower similarity with values of 0.06 and 0.09, respectively. These results suggest that species composition becomes more distinct as soil depth increases, with greater variation observed in deeper layers. The overall structure of the graph shows that micromycete communities in the upper soil layers are more similar to each other, while deeper layers contain more unique and less overlapping species compositions (Figure 4).

**Figure 5:**
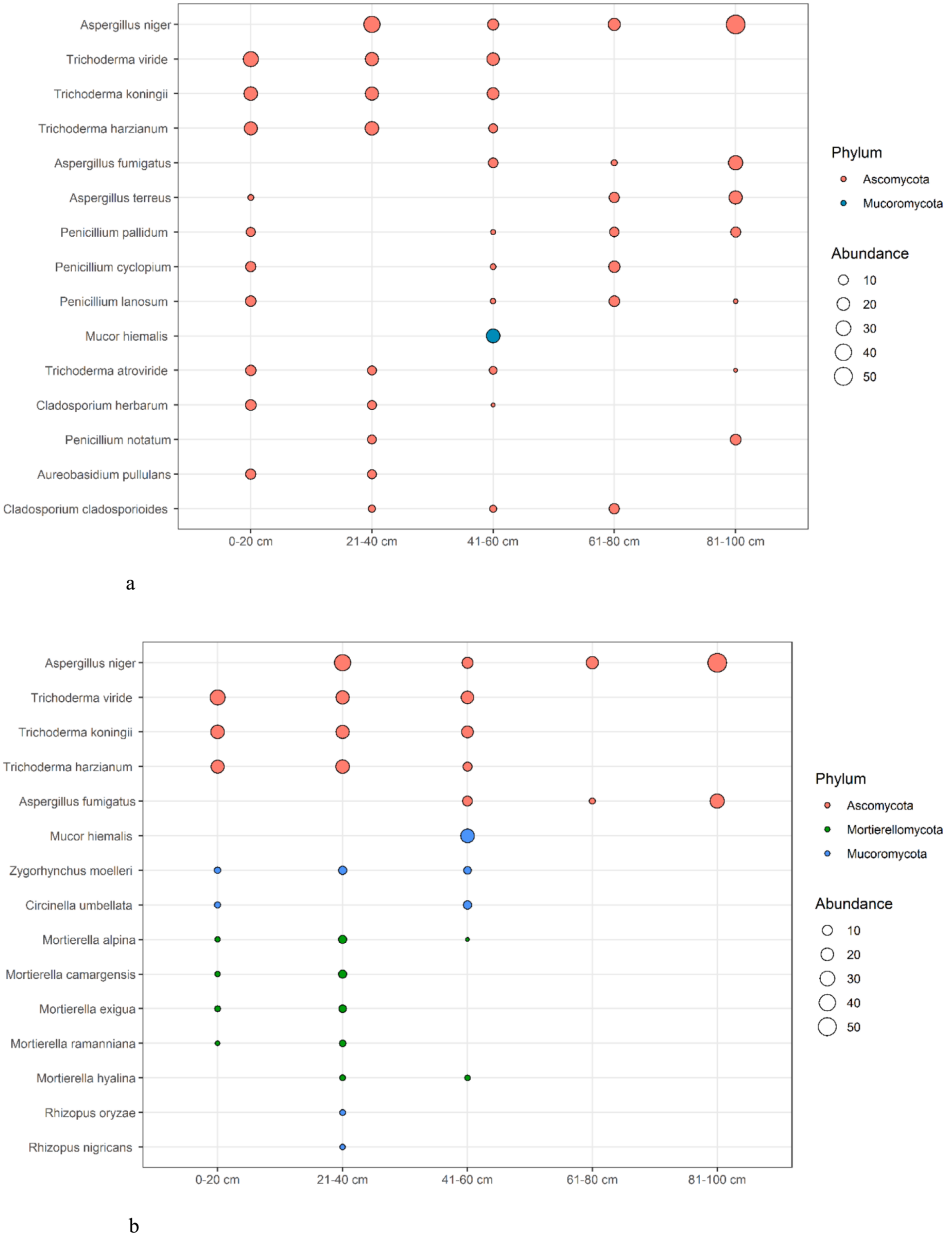
Species of the mycobiota with the highest frequency of occurrence at different soil depths (R Core Team, 2020). **(a)** Top 15 species by occurrence frequency from the entire identified fungal biodiversity. **(b)** Top-5 species of mycobiota from the Phyla Ascomycota, Mucoromycota and Mortierellomycota with the highest frequency of occurrence at different depths of soil samples.

The distribution of micromycete species at different soil depths shows that *Ascomycota* phyla dominate all layers. In particular, *Aspergillus niger* and *Trichoderma viride* show the highest abundance, both in the surface layer (0–20 cm) and at the deepest depth (80–100 cm). Other species such as *Trichoderma koningii* and *Trichoderma harzianum*, are also well represented at different depths, although their abundance is generally lower than that of *Aspergillus niger*. *Mucor hiemalis*, the only representative of the *Mucoromycota*, is predominantly found in the 40–60 cm layer, but at a significantly lower abundance compared to the dominant *Ascomycota* species. This distribution pattern suggests that *Ascomycota* species are highly adaptable to a wide range of soil depths, whereas *Mucoromycota* species are more limited in their depth range (Figure 5).

The micromycete species with the highest occurrence in different soil layers, from the phyla *Ascomycota, Mucoromycota* and *Mortierellomycota* showed a distinct distribution pattern (Figure 5).

- Top 15 species by occurrence frequency from the entire identified fungal biodiversity.
- Top-5 species of mycobiota from the Phylа *Ascomycota, Mucoromycota* and *Mortierellomycota* with the highest frequency of occurrence at different depths of soil samples.

The species from the phylum *Ascomycota*, particularly *Aspergillus niger* and *Trichoderma viride*, show the highest abundance across both surface (0–20 cm) and deeper layers (80–100 cm), indicating their wide ecological range and adaptability. In contrast, species from *Mucoromycota*, such as *Mucor hiemalis*, and *Mortierellomycota*, like *Mortierella ramanniana* and *Mortierella hyalina*, are more restricted to specific depths, primarily between 20 and 60 cm.

This suggests that while *Ascomycota* species dominate across all layers, the occurrence of *Mucoromycota* and *Mortierellomycota* is more depth-specific, reflecting their adaptation to different soil environments (Figure 5).

**Figure 6:**
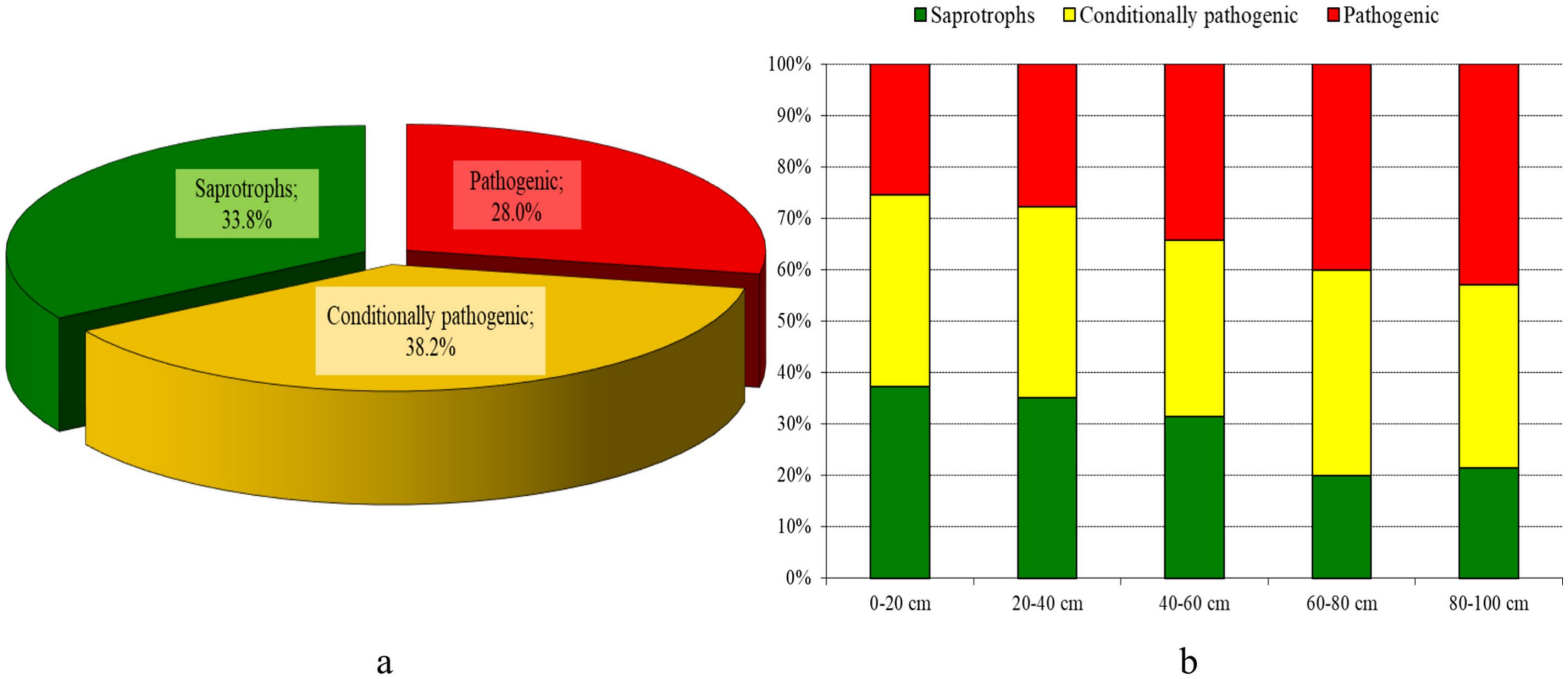
Structure of the mycobiome in the old apple orchard soil, % (**a,b**: overall structure of the mycobiome, 0–100 cm; **b**– structure of the mycobiome in different soil layers).

In the overall structure of the mycobiome of the old orchard, phytopathogens represented 28.0%, conditionally pathogenic fungi 38.2%, and saprophytic fungi made up 33.8% (Figure 6a). The diversity of phytopathogenic fungi was represented by 19 species across 12 genera. The significant proportion of phytopathogenic species indicates a high risk of disease outbreaks in apple trees and other crops. Among the identified phytopathogenic micromycetes, three species - *Botrytis cinerea*, *Fusarium oxysporum*, and *Chaetomium* spp. - are classified as some of the most dangerous worldwide, causing severe damage to crops (Venbrux et al., 2023). Consequently, monitoring these pathogens and implementing environmentally safe methods to mitigate their impact is crucial.

Most of the root rot pathogens identified are facultative parasites that persist on plant debris or directly in the soil, linking their life cycles closely to soil health. The highest percentage of pathogenic (40–43%) and conditionally pathogenic (36–40%) fungal species was found in the soil layers at depths of 60–80 cm and 80–100 cm (Figure 6b). The data further suggest a decrease in saprotroph dominance in deeper layers, correlating with the increased presence of pathogenic species, highlighting the potential risk for root diseases in young apple trees planted in these soils.

**Figure 7:**
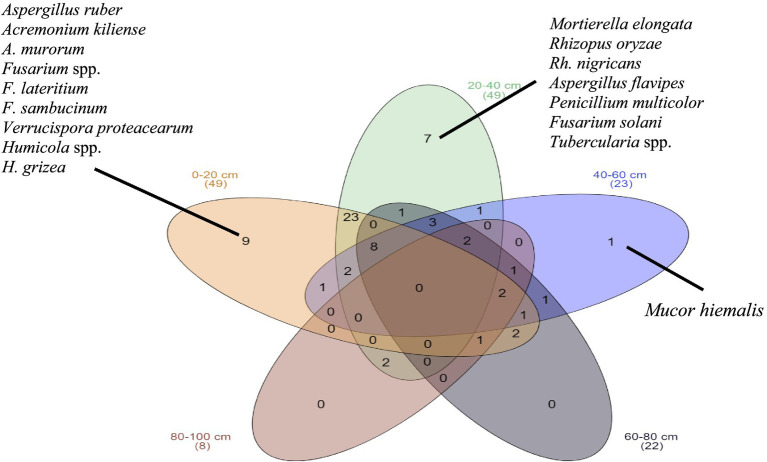
Venn’s diagram that visualizes the general mycobiota, the species that are unique to the sampling sites, and those are shared, the number of species.

To visualize the overall mycobiome of the soil, as well as the species that are unique or common to different soil layers, a Venn diagram was constructed (Figure 7).

The Venn diagram provides insights into the distribution patterns and ecological roles of micromycetes at different soil depths, showing that certain species are depth-specific while others are generalists. This information is crucial for understanding soil health, biodiversity, and the potential impact of these organisms on plant health, particularly in the context of orchard management and disease prevention strategies. Species distribution across soil depths: The diagram shows that certain species are unique to specific soil layers, indicating that different environmental conditions at each depth may favor the growth of particular micromycetes. For example, species such as *Mortierella elongata* and *Rhizopus oryzae* were found exclusively at certain depths, suggesting that these species are better adapted to the conditions at those specific soil layers. Shared species among depths: The central region of the Venn diagram indicates species that are common across multiple soil depths, reflecting the ability of certain micromycetes to thrive in a broader range of environmental conditions. The presence of shared species, such as *Aspergillus flavipes* and *Fusarium solani*, suggests that these fungi possess generalist traits that allow them to colonize multiple soil layers. Ecological implications: The fact that a significant number of species are shared between the upper and middle soil layers (0–60 cm) suggests that these layers may provide more consistent environmental conditions, such as nutrient availability or moisture, compared to the deeper layers (60–100 cm). The unique species found in the deeper layers may indicate specialized adaptations to reduced organic matter and lower oxygen availability. Mycobiome complexity: The overlapping species and the presence of both unique and shared taxa highlight the complexity of the mycobiome in long-term apple orchards. This complexity likely contributes to the stability of the soil microbial community, influencing soil health and plant disease dynamics.

## 4 Discussion

In the overall structure of the soil mycobiome of the orchard, fungi from the Phylum *Ascomycota* dominated, accounting for 85% of the species. *Ascomycota* is associated with a wide range of monoculture systems (Xiong et al., 2015) and is also dominant in the soil of many orchards (Han et al., 2023). The classes *Eurotiomycetes* (27 species, or 47%) and *Sordariomycetes* (20 species, or 35%) within *Ascomycota* are dominant, consistent with many studies that have found *Sordariomycetes* to be the most prevalent fungal class in various agricultural systems. Members of this class act as both pathogens and endophytes of plants in nearly all ecosystems (Li et al., 2014). Among the representatives of this Phylum, the highest species diversity was found in the genera *Aspergillus* (14 species) and *Penicillium* (13 species), which were present in nearly all of the studied soil layers of the apple orchard, comprising 21 and 19% of the total mycobiome, respectively. Representatives of these genera are widely distributed in agricultural soils throughout Ukraine and can be considered key contributors to the mycobiome of the studied orchard (Simonin et al., 2020; Oliferchuk et al., 2023). Among the isolated cultures from the Phylum *Ascomycota*, 1–2 species were represented from the genera *Gliocladium*, *Botrytis*, *Tubercularia*, *Alternaria*, *Aureobasidium*, *Phoma*, *Verrucispora*, *Chaetomium*, *Colletotrichum*, and *Ulocladium*, which together accounted for 16.4% of the total mycobiome. The genera *Cladosporium*, *Humicola*, and *Acremonium* were each represented by 3 species, contributing 4% each to the structure. The genera *Fusarium* and *Trichoderma* made up 9 and 6% of the total mycobiome, respectively, represented by 6 and 4 species. Fungi from the Phylum *Mucoromycota* and *Mortierellomycota* were represented by significantly less diversity of genera and species comprising 15% of the mycobiome structure. The phylum *Mucoromycota* was represented by only 5 species, accounting for just 6% of the mycobiome structure. *Rhizopus*, *Mucor*, *Zygorhynchus*, and *Circinella* were represented by 1–3 species each. The phylum *Mortierellomycota* constituted 9% of the overall mycobiome structure and was represented solely by 6 species of *Mortierella*. Representatives of the Phylum *Mucoromycota* and *Mortierellomycota* were not found at a depth of 60 cm and below; they inhabited only the upper layers of the soil.

The data on the isolated and identified genera and species of micromycetes present in the soil of the studied orchard align with our previous research and studies of the mycobiota of fruit orchard soils by other authors in various parts of the world (Avellaneda-Torres and Torres-Roja, 2015; Landinez-Torres et al., 2019; Nicola et al., 2021; Ozimek and Hanaka, 2021; Oliferchuk et al., 2023). The high prevalence of species from the genus *Mortierella* has also been noted in the soils of peach and apple orchards in the Colombian Andes region (Landinez-Torres et al., 2019). Species of *Mortierella* spp. are saprotrophic fungi and play a crucial role in the transformation of nutrients in agricultural soils (Li et al., 2018; Ozimek and Hanaka, 2021).

Some species isolated from the studied soils, particularly those from the genera *Mortierella* and *Trichoderma*, have been documented in the literature as fungi of bioprospective interest. *M. exigua* and *Trichoderma atroviride* possess the potential to act as agents for the bioremediation of heavy metals (Kacprzak and Malina, 2005). According to available data, representatives of the genus *Mortierella* can serve as producers of various fatty acids, including arachidonic acid (Kikukawa et al., 2018). Arachidonic acid, as noted in scientific literature, may inhibit the growth of pathogenic fungi from the genus *Fusarium* at certain concentrations (Guimarães and Venâncio, 2022; Podgórska-Kryszczuk et al., 2022). Therefore, the isolated *M. elongata* can theoretically be considered as an agent for biological protection against pathogens (Li et al., 2018; Ozimek and Hanaka, 2021), although further research is needed to evaluate the fungistatic and immunomodulatory effects of compounds isolated from *Mortierella* fungi, as arachidonic acid from different sources may exhibit varying effects on pathogens. There is a growing interest in the application of *Mortierella* spp. primarily due to the potential use of this genus to enhance nutrient absorption efficiency, provide positive effects in protecting crops from adverse conditions, and reduce the application of chemical fertilizers and pesticides. Additionally, the activity of *Mortierella* species isolated from wild or cultivated plants influences soil microbiota and supports the productivity of beneficial microorganisms, significantly increasing the yields of agricultural crops (Ozimek and Hanaka, 2021).

Interestingly, some fungal species that are commonly described as dominant in various soil types showed low prevalence in our studies. One example is *Mortierella* spp., which is considered the most widespread as it is dispersed by wind and rain. In our study, we identified only two species, *M. elongata* and *M. hyalina*, with a relatively low occurrence rate of 1.3–1.7% at depths of 20–40 cm and 40–60 cm. Similarly, *Fusarium* spp., which is regarded as dominant in most soils (Wakelin et al., 2008), had occurrence rates of 0.7 and 2.3% in our research. A similar observation was reported in the studies by Grządziel and Gałązka (2019).

Species of the genus *Trichoderma* are considered among the most common fungi in nature due to their resilience to various stress factors and rapid growth rates (Pagano et al., 2017). In our study, four species of *Trichoderma* with high occurrence rates (4.2–32%) were found in the upper layers of soil and down to a depth of 60 cm.

*Trichoderma* species are characterized by their antibacterial activity (Hevedy et al., 2020; Ruangwong et al., 2021). The origin of the antifungal activity of *Trichoderma* spp. is not yet fully established; however, researchers suggest two possible factors: the synthesis of a complex chitinolytic enzyme system and the production of secondary metabolites with antagonistic activity (Patil et al., 2016; Zhand et al., 2018). Some *Humicola* strains are described as producers of bioorganic fertilizers or as organisms that provide control over plant diseases (Wang et al., 2019).

Interestingly, the conditionally pathogenic and pathogenic species *Rhizopus oryzae* (20–40 cm), *Aspergillus ustus*, and *A. terreus* (0–20 cm), typical for southern chernozem soils, were detected in the surface layer of the studied soil, comprising 1.4 to 1.8% of the total species count. This phenomenon can be explained, firstly, by the availability of sufficient nutrients in the upper soil layer and climate changes toward warming. We also isolated the atypical species *Aspergillus flavus* from garden biocenoses at a depth of 20–40 cm. This species is often found in grains, peanuts, and flour and poses a danger to humans and animals due to its production of aflatoxins (Frisvad et al., 2019; Benkerroum, 2020; Caceres et al., 2020). Therefore, monitoring of this species in the studied soil is essential. The species *Fusarium culmorum* Sacc. and *Trichocladium asperum* are typical representatives of chernozem soils (common low-humus chernozem, southern chernozem); however, we detected these species at depths of 40–60 cm and 20–40 cm, respectively.

It is noteworthy to mention the dynamics of the distribution of *Aspergillus niger*. This species, which constituted 39%, was found in the upper layer of the soil, while the percentage slightly decreased at depths of 20–40 cm and 40–60 cm; however, at a depth of 80–100 cm, the occurrence of this species increased to 56%.

It is important to note that we did not find the traditional pathogens of scab, powdery mildew, moniliosis, or alternaria blight. Additionally, we did not detect any species from the genus *Glomus*, which are common representatives in the soils of fruit orchards.

The species *Colletotrichum gloeosporioides* is harmful to many agricultural plants, causing anthracnose and bitter rot in apples (Wenneker et al., 2021; Chen et al., 2022).

The pathogenic complex responsible for root rot in apple trees was represented by the following species: *Fusarium* spp., *Alternaria alternata*, *Aspergillus niger*, and *Cladosporium* spp.

## 5 Conclusion

New data has been obtained regarding the composition and distribution of microscopic fungi in the layers of dark gray podzol soil following long-term apple cultivation in Ukraine. The study revealed that over 90 years of monocultural apple cultivation has resulted in the formation of a stable complex of soil micromycetes belonging to the phyla *Mucoromycota, Mortierellomycota,* and *Ascomycota*. The taxonomic list of microscopic fungi comprises 68 species from 22 genera. Among the isolated and identified micromycetes, the greatest species diversity was found in the Phylum *Ascomycota*, particularly in the genera *Aspergillus* and *Penicillium*, which were present in almost all studied soil layers of the apple orchard, constituting 21 and 19% of the total microbiome, respectively. Among the isolated cultures, 28% of the species were identified as phytopathogenic, posing a biological threat to the apple biocenosis. Pathogenic microorganisms were detected in the soil at depths of up to 1 meter, including species such as *Aspergillus niger* and *Aspergillus fumigatus*, which weaken the plant by producing toxins and promote the spread of fungi that cause root rot in apple trees, acting as biological agents of transplant disease and soil fatigue. Among them, *Colletotrichum gloeosporioides* is particularly harmful to many agricultural plants, as it is the causative agent of anthracnose and leads to bitter rot in apples. The prevalence of phytopathogenic fungi in soil, especially under long-term monoculture cultivation, can lead to significant crop losses due to plant diseases. It is important to monitor the populations of pathogenic species and implement environmentally safe methods to reduce their impact on agroecosystems.

## Data Availability

The datasets presented in this study can be found in online repositories. The names of the repository/repositories and accession number(s) can be found in the article/supplementary material.

## Footnotes

1
http://www.mycobank.org/

2
http://www.mothur.org/wiki/Calculators

3
http://www.interactivenn.net/
